# Supplementary material for: A Novel Zinc Chelator, 1H10, Ameliorates Experimental Autoimmune Encephalomyelitis by Modulating Zinc Toxicity and AMPK Activation
Source: Int J Mol Sci. 2020 May 10;21(9):3375. doi: 10.3390/ijms21093375 (PMC7247014; doi:10.3390/ijms21093375)
Supplement: Supplementary file 1 [file ijms-21-03375-s001.zip › Supplementary Materials for ijms-782894-R1-Final.docx]

**
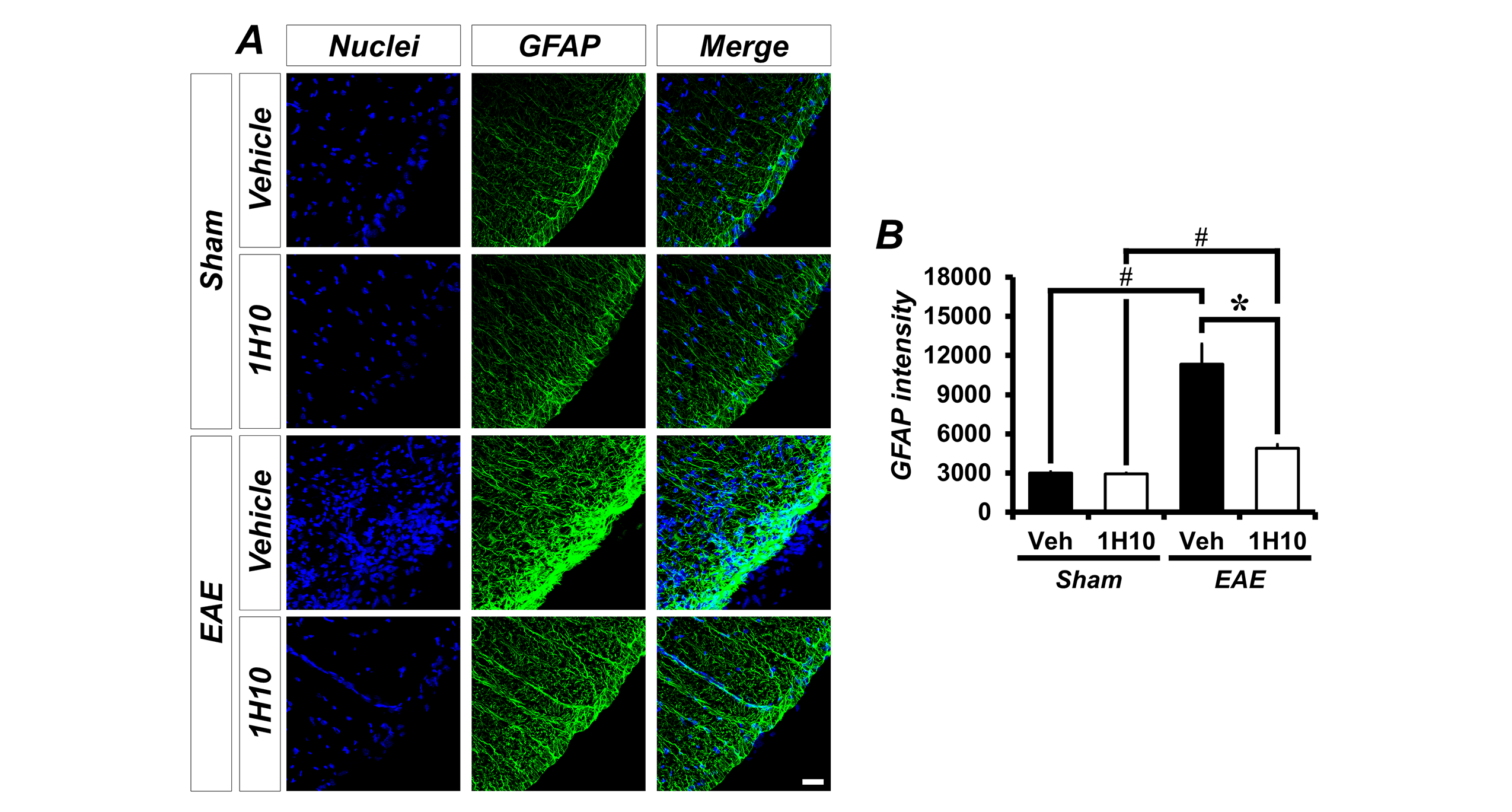
**

**Supplementary Figure 1.** 1H10 treatment reduces EAE-induced astrogliosis in the white matter of spinal cord. **(A)** Representative astrogliosis in spinal cord of sham-operated and MOG_35-55_-immunized mice (either vehicle or 1H10) at day 21 as shown by immunofluorescence for GFAP (green). Scale bar, 50 µm. **(B)** Quantification of immunofluorescence intensity of GFAP as determined in the same spinal cord region (mean ± SEM; n=4 per group). **p* < 0.05 vs. vehicle-treated EAE mice; #*p* < 0.05 vs. sham-operated mice (Kruskal–Wallis test followed by Bonferroni post-hoc test: Chi square=11.514, df=3, p=0.009).

**Supplementary Movie 1.** Behaviors of vehicle-treated control group mice.

**Supplementary Movie 2.** Behaviors of 1H10-treated control group mice.

**Supplementary Movie 3.** Behaviors of vehicle-treated EAE mice.

**Supplementary Movie 4.** Behaviors of 1H10-treated EAE mice.
