# Supplementary figures and images for: A Novel Zinc Chelator, 1H10, Ameliorates Experimental Autoimmune Encephalomyelitis by Modulating Zinc Toxicity and AMPK Activation
Source: Int J Mol Sci. 2020 May 10;21(9):3375. doi: 10.3390/ijms21093375 (PMC7247014; doi:10.3390/ijms21093375)

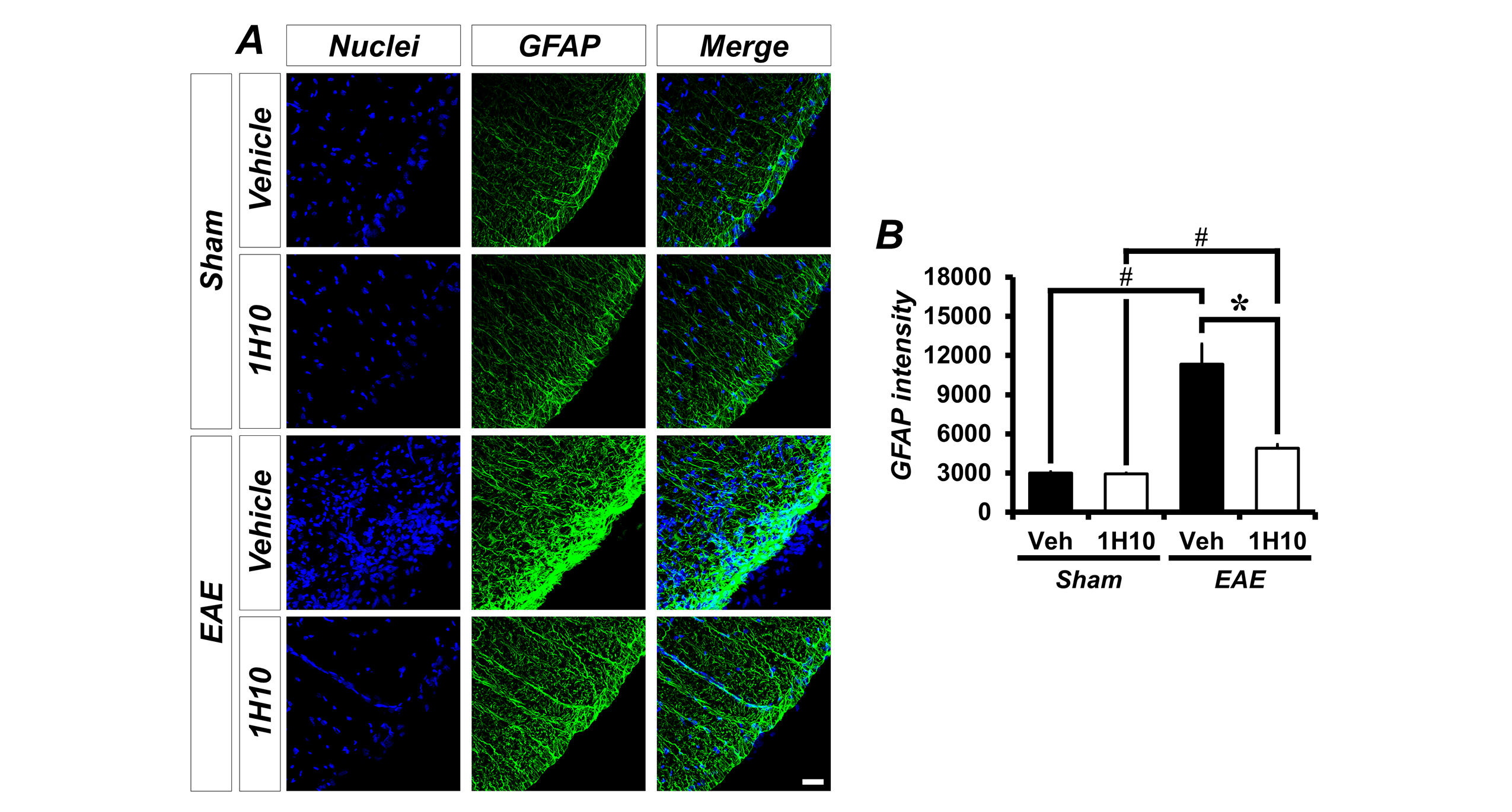

Supplement: Supplementary file 1 [file ijms-21-03375-s001.zip › Figure S1.tif]
